# Mood swings are causally associated with intracranial aneurysm subarachnoid hemorrhage: A Mendelian randomization study

**DOI:** 10.1002/brb3.3233

**Published:** 2023-08-25

**Authors:** Kang Peng, Yanwen Li, Abraham Ayodeji Adegboro, Siyi Wanggou, Xuejun Li

**Affiliations:** ^1^ Department of Neurosurgery Xiangya Hospital Central South University Changsha Hunan China; ^2^ Hunan International Scientific and Technological Cooperation Base of Brain Tumor Research Xiangya Hospital Central South University Changsha China

**Keywords:** emotion, genetics, neurosurgery, stroke

## Abstract

**Background:**

Mood swings have been observed in patients with intracranial aneurysm (IA), but it is still unknown whether mood swings can affect IA.

**Aim:**

To explore the causal association between mood swings or experiencing mood swings and IA through a two‐sample Mendelian randomization (MR) study.

**Methods:**

**S**ummary‐level statistics of mood swings, experiencing mood swings, IA, aneurysm‐associated subarachnoid hemorrhage (aSAH), and non‐ruptured IA (uIA) were collected from the genome‐wide association study. Two‐sample MR and various sensitivity analyses were employed to explore the causal association between mood swings or experiencing mood swings and IA, or aSAH, or uIA. The inverse‐variance weighted method was used as the primary method.

**Results:**

Genetically determined mood swings (odds ratio [OR] = 5.23, 95% confidence interval (95%CI): 1.65–16.64, *p* = .005) and experiencing mood swings (OR = 2.50, 95%CI: 1.37–4.57, *p* = .003) were causally associated with an increased risk of IA. Mood swings (OR = 5.67, 95%CI: 1.40–23.04, *p* = .015) and experiencing mood swings were causally associated with the risk of aSAH (OR = 2.91, 95%CI: 1.47–5.75, *p* = .002). Neither mood swings (OR = 1.95, 95%CI: .31–12.29, *p* = .478) nor experiencing mood swings (OR = 1.20, 95%CI: .48–3.03, *p* = .693) were associated with uIA.

**Conclusions:**

Mood swings and experiencing mood swings increased the risk of IA and aSAH incidence. These results suggest that alleviating mood swings may reduce IA rupture incidence and aSAH incidence.

## Graphical abstract

Mood swings are casually associated with aSAH

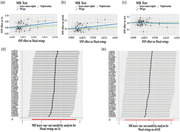

## 1 INTRODUCTION

Intracranial aneurysm (IA) is a cerebrovascular structural disease with a risk of rupture (Juvela, 2011). In the general population, the prevalence of IA is approximately 3%, and the annual risk of IA rupture is approximately 1% (Juvela, 2011; Wermer et al., 2007). Once an IA ruptures, blood enters the subarachnoid space, causing up to 30% of patients to die within the first few days, and about 50% of the survivors exhibiting significant functional disability (Nieuwkamp et al., 2009; Wiebers et al., 2003). It is critical to intervene in IA before its rupture to improve the outcome for IA patients. Hence, it is essential to gain sufficient knowledge about the occurrence of IA and reduce the risk of IA rupture.

Mood swings mean sudden or drastic changes in mood. These changes could have a positive or disruptive effect (Mandavilli, 2004). Severe mood swings could be categorized as a mental disease, such as bipolar disorder (BD), the main features of which are disability or disruptive mood (Ciullo et al., 2022; Ketter, 2010; Mandavilli, 2004). Several observational studies demonstrated that BD could increase the risk of all types of stroke incidence and mortality (Wu et al., 2013; Yuan et al., 2022). A comparative study revealed that mood swings could broaden early pulse pressure, which could result in cardiovascular and cerebrovascular risks (McGowan et al., 2021). Furthermore, a recent Mendelian randomization (MR) study provided evidence that insomnia has a causal association with IA, suggesting that mood instability probably affects IA (Peng et al., 2021). However, there is still no sufficient evidence as to whether or not mood swings can cause the formation of IAs or the occurrence of aneurysm‐associated subarachnoid hemorrhage (aSAH).

MR study is a method that allows the use of genetic variants as instrumental variables (IVs) to explore the causal association between exposure and outcome (Smith & Ebrahim, 2003). The advantage of MR is less residual confounding and reverse causation than traditional observational study (Smith & Ebrahim, 2003). Therefore, we employed two‐sample MR analysis to explore whether mood swings could influence IA by using summary data from the genome‐wide association study (GWAS). In addition, we further evaluated the association between mood swing and the rupture of IA, or non‐ruptured IA (uIA).

## 2 METHODS

### 2.1 Data availability

GWAS summary data were used for MR analysis in this study. All data acquired from IEU OpenGWAS project (https://gwas.mrcieu.ac.uk/) or the original studies. All studies were ethically approved and participants informed.

### 2.2 Exposure data

We retrieved mood swings‐related GWAS summary‐level data of single‐nucleotide polymorphisms (SNPs) in IEU open GWAS project (Table 1). We selected those SNPs related with exposure under the significant threshold (*p* < 5 × 10−8) as candidate IVs. Then, we used the linkage disequilibrium to assess the independence of the candidate IVs (*r* ^2^ = .001, kb = 10,000), and excluded the dependent candidate IVs (Chang et al., 2015). We further used the harmonization effect to remove palindromic SNPs and align the effect alleles of the IVs. Additionally, we calculated the *F*‐statistic, which reflected the strength of association between IVs and exposure (Burgess et al., 2011). For the SNPs, *F*‐statistics less than 10 were excluded to ensure adequate power of IVs on exposure.

**TABLE 1:** Characteristics of genome‐wide association study (GWAS) data.

GWAS	GWAS ID	Phenotypes	Used as	Sample size (*n*)	Ancestry
Ben et al.	ukb‐b‐14180	Mood swings	Exposure	204,412 cases/247,207 controls	European
Nagel et al.	ebi‐a‐GCST006944	Experiencing mood swings	Exposure	373,733^a^	European
Bakker et al.	NA	IA	Outcome	7495 cases/71,934 controls	European
	NA	aSAH	Outcome	5140 cases/71,934 controls	European
	NA	uIA	Outcome	2070 cases/71,934 controls	European

Abbreviations: aSAH, aneurysm‐associated subarachnoid hemorrhage; IA, intracranial aneurysm; NA, not applicable; uIA, non‐ruptured intracranial aneurysm.

^a^ Number of the total sample size.

### 2.3 Outcome data

We obtained the GWAS summary data of IAs from the stage 1 of a published study which consisted of 7495 cases (including: 5140 aSAH cases, 2070 uIA cases, and 285 IA cases of unknown status) and 71,934 controls (Bakker et al., 2020). The ancestry of individual in the stage 1 is European.

### 2.4 Statistical analysis

All statistical analyses were carried out using “Two Sample MR,” “MR‐PRESSO (Mendelian Randomization Pleiotropy Residual Sum and Outlier)” packages in RStudio (version 4.2.1). Inverse‐variance weighted regression analysis (IVW) was used as the primary MR analysis in this study. The IVW assumes there are no invalid instruments (Burgess et al., 2017). The effect of exposure on outcome was expressed as ratio (Wald estimate). The Cochran *Q* test was performed to examine the heterogeneity between two different genetic variants. When heterogeneity existed, we performed MR‐PRESSO for the detection and removal of outliers to correct horizontal pleiotropy (Verbanck et al., 2018). MR‐Egger regression analysis was conducted to evaluate the directional pleiotropy of IV to examine whether it violated the MR assumptions (Burgess & Thompson, 2017). Additionally, leave‐one‐out sensitivity analysis was performed to assess the stability of the MR results.

All results were expressed as odds ratios (ORs) and corresponding 95% confidence intervals (95%CIs) in this study. Statistically significant difference was considered when *p* <  .05.

## 3 RESULTS

### 3.1 Mood swings were causally and positively associated with the risk of IA and aSAH, but not non‐ruptured IAs

**FIGURE 1:**
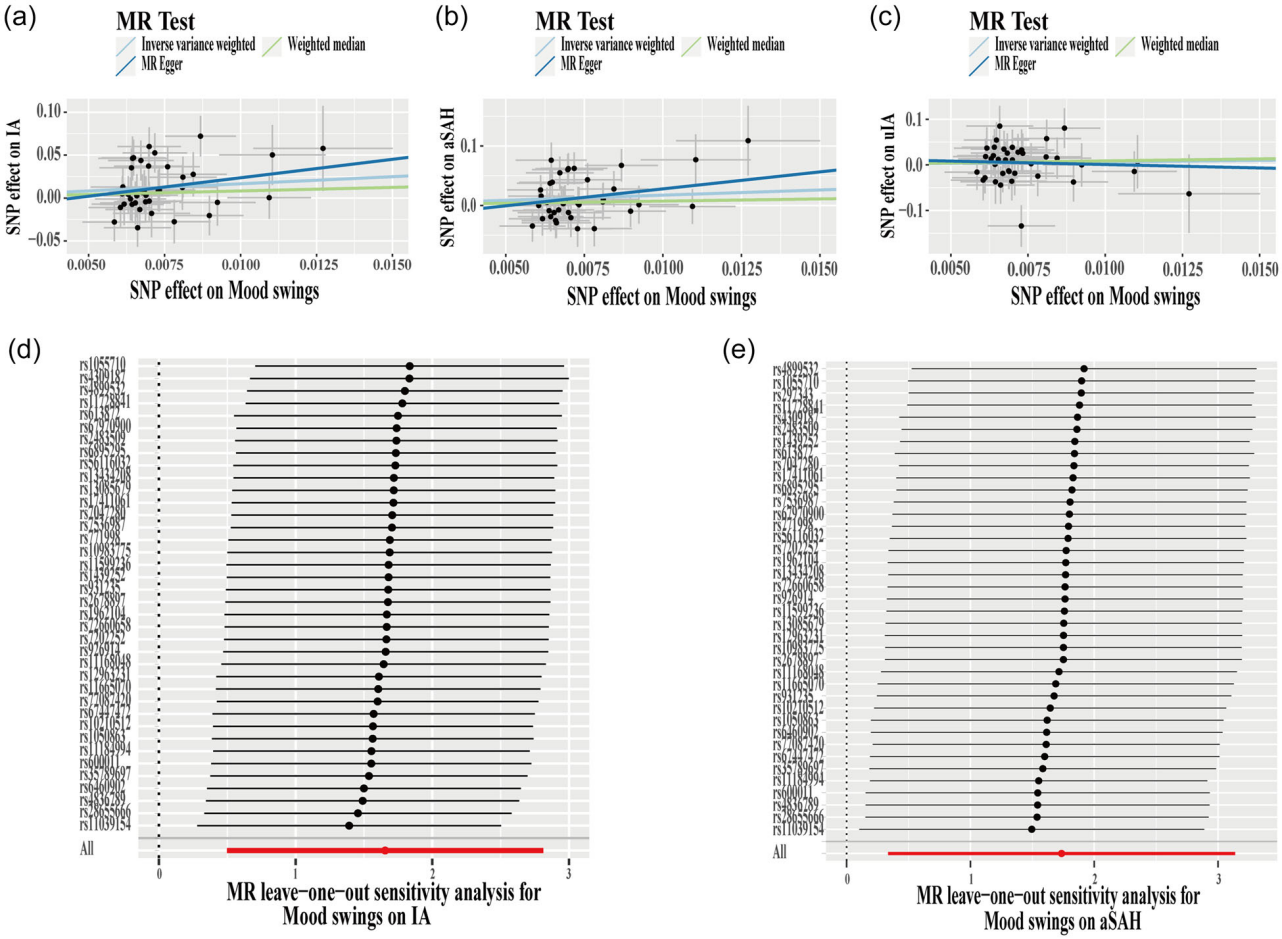
Mendelian randomization (MR) analysis of mood swings on intracranial aneurysm (IA), or aneurysm‐associated subarachnoid hemorrhage (aSAH), or non‐ruptured IA (uIA). Scatter plot of MR analysis for mood swings on IA (a), aSAH (b), and uIA (c). Leave‐one‐out sensitivity analysis of mood swings on IA (d) and aSAH (a).

We first performed two‐sample MR analysis to detect the association between mood swings and total IA cases. And we found 39 SNPs as candidate IVs (Table S1). Among these SNPs, one SNP (rs297343) showed heterogeneity following Cochran's *Q* test; then we removed the SNP using the MR‐PRESSO outlier test. Thus, a total of 38 effective IVs were used to conduct MR analysis (Table 2). The IVW result showed that mood swings were causally and positively associated with increased risk of IA (OR = 5.23, 95%CI: 1.65–16.64, *p* = .005; Table 2 and Figure 1a). MR‐Egger regression showed that there is no directional pleiotropy (*p* = .468; Table 2). To classify the overall stability of MR analysis results, leave‐one‐out analysis was employed. In addition, there was no specific IV which could lead to instability of MR results (Figure 1d).

**TABLE 2:** Inverse‐variance weighted estimates for the genetic casual association between exposure (mood swings) and outcomes (intracranial aneurysm [IA]/aneurysm‐associated subarachnoid hemorrhage [aSAH]/non‐ruptured IA [uIA]).

Exposure	Outcome	SNPs	OR (95%CI)	*p*‐Value	*p*‐Egger	*p*‐het
Mood swings	IA	38	5.23 (1.65–16.64)	.005	.468	.163
	aSAH	39	5.67 (1.40–23.04)	.015	.376	.076
	uIA	39	1.95 (.31–12.29)	.478	.705	.766

Abbreviations: CI, confidence interval; OR, odd ratio; *p*‐Egger, *p‐*value of the Egger intercept; *p*‐het, *p‐*value of the Cochran's *Q* test; *p*‐value, *p‐*value of the inverse‐variance weighted regression analysis; SNP, single nucleotide polymorphism.

To further explore whether mood swings affect IA formation or risk of rupture of IA, we performed MR analysis of mood swings on the aSAH group and the uIA group. Similar to the IAs, 39 SNPs were found as candidate IVs (Table S1), and there was no heterogeneity (aSAH: *p* = .076; uIA: *p* = .766; Table 2) and directional pleiotropy (aSAH: *p* = .376; uIA: *p* = .705; Table 2). All 39 SNPs were used to perform MR analysis. The IVW results suggested that mood swings were causally and positively associated with increased risk of the aSAH (OR = 5.67, 95%CI: 1.40–23.04, *p* = .015; Table 2 and Figure 1b), but not with the uIA (OR = 1.95, 95%CI: .31–12.29, *p* = .478; Table 2 and Figure 1c). Leave‐one‐out analysis showed that there was no SNP that affected the stability of mood swings‐aSAH MR results (Figure 1e).

### 3.2 Experiencing mood swings was also causally and positively associated with the risk of IA and aSAH

**FIGURE 2:**
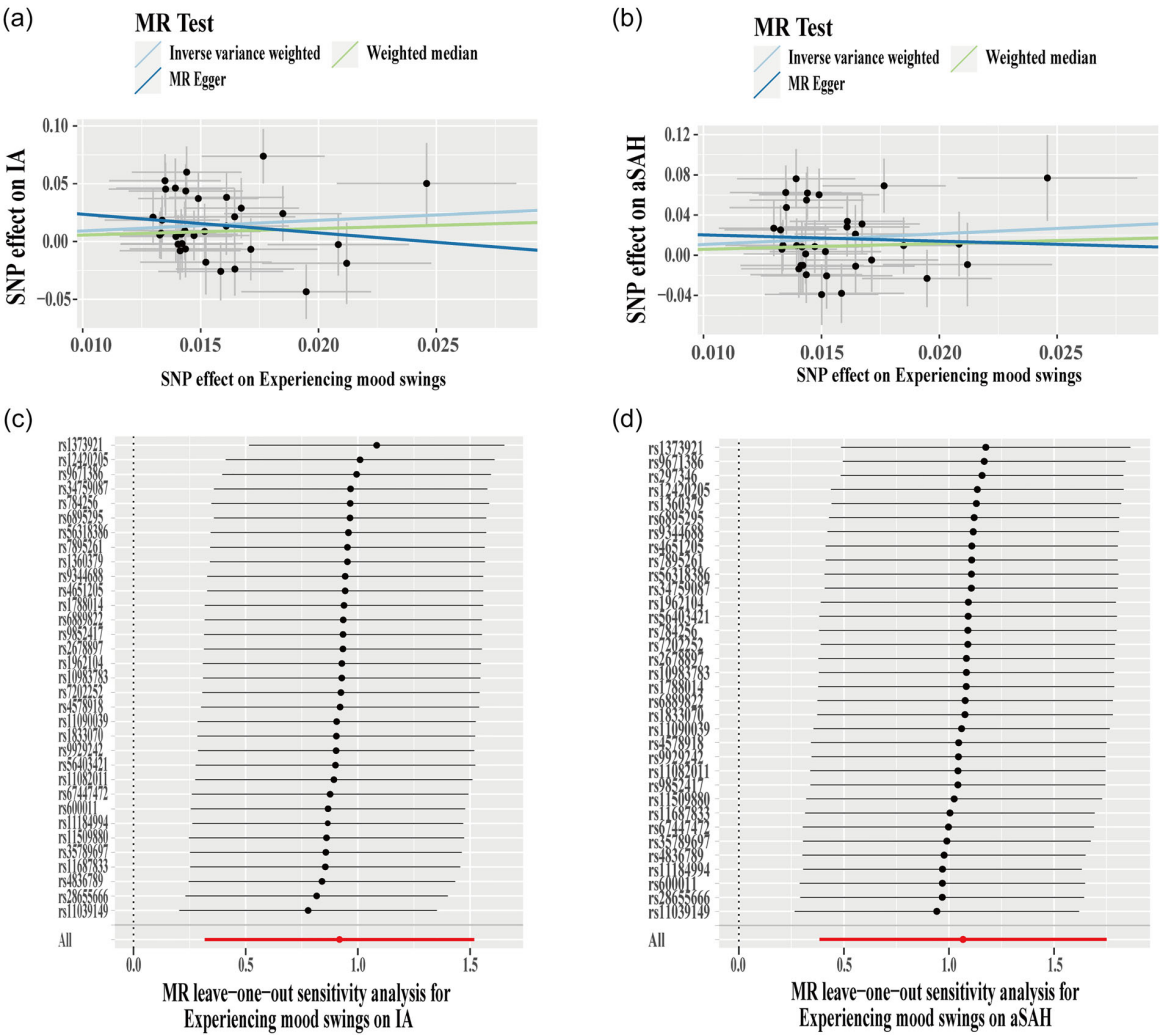
Mendelian randomization (MR) analysis of experiencing mood swings on intracranial aneurysm (IA) and aneurysm‐associated subarachnoid hemorrhage (aSAH). Scatter plot of MR analysis of experiencing mood swings on IA (a) and aSAH (b). Leave‐one‐out sensitivity analysis of experiencing mood swings on IA (c) and aSAH (d).

In addition to exploring the causal association between mood swings and the three groups (IA, aSAH, and uIA), we also investigated whether experiencing mood swings was causally associated with the three groups. Thirty‐four SNPs were found as candidate IVs (Table S2). For the MR analysis of experiencing mood swings on the IA group, one SNP (rs297346) was excluded due to heterogeneity by using the MR‐PRESSO outlier test. Thus, 33 SNPs were used to perform MR analysis of experiencing mood swings on the IA group, and 34 SNPs were used to perform MR analysis of experiencing mood swings on the aSAH group and the uIA group (Table 3). There was no heterogeneity (IA: *p* = .127; aSAH: *p* = .164; uIA: *p* = .585; Table 3) and directional pleiotropy (IA: *p* = .233; aSAH: *p* = .499; uIA: *p* = .285; Table 3). The IVW results showed that experiencing mood swings was causally associated with increased risk of the IA (OR = 2.50, 95%CI: 1.37–4.57, *p* = .003; Table 3 and Figure 2a) and increased risk of the aSAH (OR = 2.91, 95%CI: 1.47–5.75, *p* = .002; Table 3 and Figure 2b), but not with the uIA (OR = 1.20, 95%CI: .48–3.03, *p* = .693; Table 3). In addition, the leave‐one‐out analysis showed that the MR results of experiencing mood swings on the IA group (Figure 2c) and the aSAH group (Figure 2d) were stable.

**TABLE 3:** Inverse‐variance weighted estimates for the genetic casual association between exposure (experiencing mood swings) and outcomes (intracranial aneurysm [IA]/aneurysm‐associated subarachnoid hemorrhage [aSAH]/non‐ruptured IA [uIA]).

Exposure	Outcome	SNPs	OR (95%CI)	*p*‐Value	*p*‐Egger	*p*‐het
Experiencing mood swings	IA	33	2.50 (1.37–4.57)	.003	.233	.127
	aSAH	34	2.91 (1.47–5.75)	.002	.499	.164
	uIA	34	1.20 (.48–3.03)	.693	.285	.585

Abbreviations: CI, confidence interval; OR, odd ratio; *p*‐Egger, *p‐*value of the Egger intercept; *p*‐het, *p‐*value of the Cochran's *Q* test; *p*‐value, *p‐*value of the inverse‐variance weighted regression analysis; SNP, single nucleotide polymorphism.

## 4 DISCUSSION

In this study, we investigated the causal relationship between mood swings or experiencing mood swings and IA based on the European population, using two‐sample MR analysis. Our study provides evidence that genetically determined and experiencing mood swings are causally and positively correlated with the risk of IA and further confirms that mood swings and experiencing mood swings are causally and positively related to the risk of rupture of IA but not causally related to the formation of uIA.

Some meta‐analytical studies illustrated that severe mental illness (e.g., schizophrenia, BD) was associated with higher incidence of cardiocerebral vascular disease (Lambert et al., 2022). In addition, positive psychological health could reduce the incidence of stroke (Lambiase et al., 2015). A recent MR analysis demonstrated that genetically determined mood swings were causally related to the risk of intracerebral hemorrhage (ICH) (Wang et al., 2022). IA and aSAH share up to 50% genetic characteristics with ICH (Bakker et al., 2020; Mayerhofer et al., 2022; Tromp et al., 2014; Wahab et al., 2019; Yamada et al., 2018). The overlapping genes provided the avenue to explore the exposures that were relevant to ICH in IA/SAH. And another MR analysis suggested that mood instability was probably associated with IA (Peng et al., 2021). A previous study observed that anxiety and depression of patients with IAs were increased, especially those patients who experienced aSAH and still harbored an uIA (King et al., 2005). A cross‐sectional study also demonstrated that the emotional symptoms of patients with uIA could have negative influences on their health‐related quality of life (Lemos et al., 2020). In contrast, several other studies did not find any apparent influence on mood after patients were diagnosed with uIA (Buijs et al., 2012; Li et al., 2017). Such discrepant results may be owed to the fact that different questionnaires were used to assess the psychological condition of the patients. However, another observational study showed that stress was related to the increased risk of uIA and aSAH (de Wilde et al., 2019). In our study, we extracted summary‐level statistics of mood swings and experiencing mood swings from the publicly available GWAS data, and proved that genetically determined mood swings and experiencing mood swings could result in the rupture of IA and aSAH formation. Due to the distinct clinical prognosis of aSAH and uIA, alleviating mood swings may reduce the rupture risk of IA, especially those uIA patients who prefer long‐term, follow‐up observation or waiting for surgical intervention.

Our findings contribute new knowledge to the field by demonstrating a causal association between mood swings and experiencing mood swings with IA/aSAH. Subsequent studies will focus on the specific mechanism that mediates the causal association and pharmacological treatments to ease the mood swings. Previous studies have classified hypertension as a risk factor of aSAH (Feigin et al., 2005; Sundstrom et al., 2019). It has also been shown that mood swings may result in elevated pulse pressure (McGowan et al., 2021). Oxidative stress has been reported to be related to psychiatric traits (Kim et al., 2019; Lu et al., 2022). Oxidative stress and inflammation are known to play a critical role in the development and rupture of IAs (Chalouhi et al., 2012, 2013; Hasan et al., 2012; Hosaka & Hoh, 2014). Mood swings could decrease the level of total serum bilirubin, which could be associated with hypertension (Fu et al., 2021). Increased serum inflammatory biomarkers, such as cyclooxygenase 2, arachidonic acid, interleukin‐6 (IL‐6), and tumor necrosis factor alpha (TNF‐α), have been observed in individuals with mood swings (Bavaresco et al., 2020). An earlier study showed that the serum level of IL‐6 was increased in estrogen‐deficient mice, promoting estrogen deficiency‐associated IA rupture by macrophage infiltration to the wall of IA (Wajima et al., 2020). In the animal model of IA, the expression of TNF‐a was upregulated, and blocking TNF‐α could reduce the incidence of IA formation and rupture (Starke et al., 2014). It has been shown that the gut microbiota could affect the progression and prognosis of ischemic stroke and atherosclerosis through the modulation of metabolic and immunoregulatory axes in the animal model (Backhed, 2013; Benakis et al., 2016; Holmes et al., 2012; Ridler, 2016). The gut microbiota has been reported to potentially affect the formation of IA and its rupture (Chalouhi et al., 2013; Shikata et al., 2019). Depleting the gut microbiota using antibiotic treatment could reduce the incidence of the formation of IA in mouse models (Shikata et al., 2019). A recent review summarized that there was a potential link between mood swings and the gut microbiota (Obi‐Azuike et al., 2023). Thus, mood swings may possibly increase the risk of the formation of IA and its rupture through changing the gut microbiota composition. A multicenter retrospective study showed that increased body mass index was associated with the risk of IA rupture in male patients aged beyond 50‐year old (Chen et al., 2021). Emotional eating was suggested to contribute to the development of obesity and abdominal obesity (Konttinen et al., 2019). However, the complicated mechanisms of the association between mood swings and IA still warrant further exploration.

There are several strengths in our study, including the use of publicly available summary‐level statistics with a large sample size, and the utility of multiple sensitivity analyses to assess the stability of our results. This study, however, is not without limitations. First, only the European population was included. The results of this study may not be suitable for non‐European populations, and further research is needed in other populations. Furthermore, due to the use of summary‐level data, sample overlap between exposure and outcome cannot be resolved. Third, the principle of MR analysis is based on disease‐related SNP, further research is needed to establish other genetical associations between mood swings and IA/aSAH utilizing proper methods. Finally, MR analysis assesses lifelong effects rather than acute effects. For the risk of rupture of IA, acute effects may play a more crucial role.

## 5 CONCLUSIONS

In this MR study, we provided evidence that genetically determined mood swings and experiencing mood swings were causally associated with IA and aSAH. These results indicate that alleviating mood swings may help prevent IA rupture and aSAH incidence.

## AUTHOR CONTRIBUTIONS

Kang Peng collected the data and performed the analyses. Kang Peng, Yanwen Li, and Abraham Ayodeji Adegboro wrote the manuscript. Xuejun Li and Siyi Wanggou conceived the original idea and supervised the project. All authors read and approved the final manuscript.

## CONFLICT OF INTEREST STATEMENT

The authors declared there were no any conflicts of interest.

### Peer Review

The peer review history for this article is available at https://publons.com/publon/10.1002/brb3.3233.

## Supporting information

Table S1 Thirty nine valid IVs were used for MR analysis of mood swings on IA, or aSAH, or uIA.
Table S2 Thirty‐four valid IVs were used for MR analysis of experiencing mood swings on IA, or aSAH, or uIA.

## Data Availability

All data acquired from IEU OpenGWAS project (https://gwas.mrcieu.ac.uk/) or the original studies. And All data in this article can be provided by the corresponding author Dr. Xuejun Li.
