# Supplementary material for: Mood swings are causally associated with intracranial aneurysm subarachnoid hemorrhage: A Mendelian randomization study
Source: Brain Behav. 2023 Aug 25;13(11):e3233. doi: 10.1002/brb3.3233 (PMC10636415; doi:10.1002/brb3.3233)
Supplement: Supplementary file 1 — Table S1 Thirty nine valid IVs were used for MR analysis of mood swings on IA, or aSAH, or uIA. Table S2 Thirty‐four valid IVs were used for MR analysis of experiencing mood swings on IA, or aSAH, or uIA. [file BRB3-13-e3233-s001.docx]

**Table.S1. 39 valid IVs were used for MR analysis of mood swings on IA, or aSAH, or uIA.**

| SNP | effect allele | non-effect allele | effect allele frequency | SE | BETA | p-value | F-statistics |
| --- | --- | --- | --- | --- | --- | --- | --- |
| rs10210512 | G | T | 0.4201 | 0.0011 | 0.0064 | 1.30E-09 | 36.80 |
| rs1050863 | A | G | 0.5666 | 0.0011 | -0.0076 | 5.80E-13 | 51.90 |
| rs1055710 | A | G | 0.3317 | 0.0011 | -0.0066 | 2.30E-09 | 35.67 |
| rs10983775 | T | C | 0.5344 | 0.0010 | -0.0069 | 5.10E-11 | 43.14 |
| rs11039154 | T | C | 0.2776 | 0.0012 | 0.0087 | 8.80E-14 | 55.63 |
| rs11168048 | C | T | 0.4190 | 0.0011 | -0.0061 | 6.30E-09 | 33.75 |
| rs11184994 | T | C | 0.6941 | 0.0011 | 0.0064 | 1.20E-08 | 32.53 |
| rs11599236 | C | T | 0.4111 | 0.0011 | -0.0063 | 3.60E-09 | 34.83 |
| rs11665070 | A | G | 0.6671 | 0.0011 | -0.0084 | 2.60E-14 | 58.01 |
| rs11728841 | C | A | 0.5558 | 0.0011 | 0.0058 | 2.90E-08 | 30.81 |
| rs12963231 | A | C | 0.3294 | 0.0011 | 0.0081 | 3.50E-13 | 52.90 |
| rs13085679 | A | G | 0.5007 | 0.0010 | 0.0069 | 4.20E-11 | 43.52 |
| rs13434208 | G | A | 0.5251 | 0.0010 | -0.0060 | 7.60E-09 | 33.39 |
| rs1439252 | A | G | 0.3732 | 0.0011 | -0.0066 | 1.30E-09 | 36.83 |
| rs17411061 | T | C | 0.4195 | 0.0011 | 0.0070 | 4.10E-11 | 43.56 |
| rs1962104 | C | T | 0.5581 | 0.0011 | 0.0073 | 5.50E-12 | 47.49 |
| rs2483509 | A | G | 0.4499 | 0.0010 | 0.0064 | 8.40E-10 | 37.67 |
| rs2678897 | A | G | 0.6075 | 0.0011 | 0.0068 | 2.50E-10 | 40.04 |
| rs28655666 | A | G | 0.5523 | 0.0010 | -0.0070 | 2.40E-11 | 44.65 |
| rs297343* | G | T | 0.6378 | 0.0011 | -0.0073 | 2.10E-11 | 44.83 |
| rs35789697 | A | G | 0.3694 | 0.0011 | -0.0067 | 5.00E-10 | 38.68 |
| rs4309187 | C | A | 0.6821 | 0.0011 | 0.0090 | 1.40E-15 | 63.74 |
| rs4836789 | C | T | 0.5963 | 0.0011 | 0.0072 | 1.50E-11 | 45.56 |
| rs4899532 | G | A | 0.7452 | 0.0012 | 0.0078 | 6.40E-11 | 42.69 |
| rs56116032 | G | A | 0.2194 | 0.0013 | -0.0092 | 2.40E-13 | 53.63 |
| rs600011 | C | A | 0.3014 | 0.0011 | 0.0070 | 8.40E-10 | 37.66 |
| rs613872 | T | G | 0.8258 | 0.0014 | 0.0109 | 1.80E-15 | 63.31 |
| rs6460902 | A | G | 0.4190 | 0.0011 | 0.0065 | 8.10E-10 | 37.74 |
| rs67447472 | T | G | 0.0996 | 0.0018 | 0.0110 | 2.80E-10 | 39.80 |
| rs67970900 | T | G | 0.3088 | 0.0011 | -0.0067 | 3.20E-09 | 35.06 |
| rs6895295 | T | C | 0.2157 | 0.0013 | 0.0071 | 2.60E-08 | 30.99 |
| rs7047280 | T | C | 0.6050 | 0.0011 | -0.0062 | 7.80E-09 | 33.33 |
| rs7202252 | C | T | 0.7324 | 0.0012 | 0.0073 | 4.90E-10 | 38.71 |
| rs72660658 | T | C | 0.2672 | 0.0012 | -0.0068 | 6.90E-09 | 33.56 |
| rs7536987 | G | A | 0.2775 | 0.0012 | -0.0065 | 1.90E-08 | 31.55 |
| rs77087420 | G | A | 0.0546 | 0.0023 | -0.0127 | 3.00E-08 | 30.72 |
| rs771998 | C | T | 0.2763 | 0.0012 | 0.0064 | 4.80E-08 | 29.78 |
| rs926914 | T | C | 0.2884 | 0.0012 | 0.0081 | 2.30E-12 | 49.17 |
| rs931235 | C | A | 0.5929 | 0.0011 | -0.0061 | 9.40E-09 | 32.97 |

The SNP with * was removed in the MR analysis of mood swings on IA.

**Table.S2 34 valid IVs were used for MR analysis of experiencing mood swings on IA, or aSAH, or uIA.**

| SNP | effect allele | non-effect allele | effect allele frequency | SE | BETA | p-value | F-statistics |
| --- | --- | --- | --- | --- | --- | --- | --- |
| rs10983783 | T | G | 0.5533 | 0.0023 | -0.0133 | 1.04E-08 | 32.76 |
| rs11039149 | G | A | 0.2695 | 0.0026 | 0.0177 | 1.29E-11 | 45.83 |
| rs11082011 | T | C | 0.6560 | 0.0024 | -0.0167 | 8.74E-12 | 46.59 |
| rs11090039 | A | G | 0.2833 | 0.0026 | 0.0164 | 1.75E-10 | 40.73 |
| rs11184994 | T | C | 0.6915 | 0.0025 | 0.0139 | 2.82E-08 | 30.84 |
| rs11509880 | A | G | 0.3442 | 0.0024 | 0.0161 | 4.18E-11 | 43.53 |
| rs11687833 | T | C | 0.3941 | 0.0024 | 0.0135 | 2.06E-08 | 31.44 |
| rs12420205 | T | C | 0.6775 | 0.0025 | 0.0165 | 4.77E-11 | 43.27 |
| rs1360379 | T | C | 0.6044 | 0.0024 | -0.0144 | 1.31E-09 | 36.8 |
| rs1373921 | G | A | 0.2303 | 0.0028 | 0.0195 | 1.41E-12 | 50.17 |
| rs1788014 | G | A | 0.4431 | 0.0024 | 0.0147 | 4.07E-10 | 39.08 |
| rs1833070 | A | G | 0.3612 | 0.0024 | -0.0134 | 4.20E-08 | 30.05 |
| rs1962104 | C | T | 0.5504 | 0.0024 | 0.0152 | 1.38E-10 | 41.19 |
| rs2678897 | A | G | 0.6114 | 0.0024 | 0.0142 | 3.12E-09 | 35.11 |
| rs28655666 | A | G | 0.5443 | 0.0023 | -0.0144 | 5.66E-10 | 38.44 |
| rs297346* | G | A | 0.6339 | 0.0024 | -0.0150 | 4.58E-10 | 38.85 |
| rs34759087 | T | C | 0.1178 | 0.0036 | -0.0212 | 3.53E-09 | 34.87 |
| rs35789697 | A | G | 0.3582 | 0.0024 | -0.0144 | 2.82E-09 | 35.31 |
| rs4578918 | C | T | 0.7432 | 0.0027 | -0.0161 | 1.38E-09 | 36.69 |
| rs4651205 | T | C | 0.2802 | 0.0026 | -0.0142 | 3.91E-08 | 30.2 |
| rs4836789 | C | T | 0.5971 | 0.0024 | 0.0135 | 1.15E-08 | 32.58 |
| rs56318386 | T | C | 0.2152 | 0.0028 | -0.0171 | 1.37E-09 | 36.71 |
| rs56403421 | C | A | 0.3270 | 0.0025 | 0.0185 | 1.21E-13 | 55 |
| rs600011 | C | A | 0.2922 | 0.0026 | 0.0149 | 5.38E-09 | 34.05 |
| rs67447472 | T | G | 0.1042 | 0.0038 | 0.0246 | 1.06E-10 | 41.71 |
| rs6889822 | G | A | 0.3857 | 0.0024 | -0.0139 | 4.66E-09 | 34.33 |
| rs6895295 | T | C | 0.2237 | 0.0028 | 0.0152 | 4.86E-08 | 29.78 |
| rs7202252 | C | T | 0.7334 | 0.0026 | 0.0143 | 4.75E-08 | 29.81 |
| rs784256 | A | G | 0.8150 | 0.0030 | 0.0208 | 3.70E-12 | 48.27 |
| rs7895261 | G | T | 0.2900 | 0.0026 | -0.0141 | 3.70E-08 | 30.31 |
| rs9344688 | G | A | 0.3518 | 0.0024 | -0.0140 | 8.97E-09 | 33.05 |
| rs9671386 | A | G | 0.7438 | 0.0027 | 0.0159 | 2.24E-09 | 35.75 |
| rs9852417 | A | C | 0.5985 | 0.0024 | -0.0133 | 2.42E-08 | 31.13 |
| rs9929242 | A | G | 0.5288 | 0.0023 | -0.0130 | 2.57E-08 | 31 |

The SNP with * was removed in the MR analysis of experiencing mood swings on IA.
